# Learning Electronic Polarizations in Aqueous Systems

**DOI:** 10.1021/acs.jcim.4c00421

**Published:** 2024-05-28

**Authors:** Arnab Jana, Sam Shepherd, Yair Litman, David M. Wilkins

**Affiliations:** † Centre for Quantum Materials and Technologies, School of Mathematics and Physics, Queen’s University Belfast, Belfast BT7 1NN, U.K.; ‡ Yusuf Hamied Department of Chemistry, University of Cambridge, Lensfield Road, Cambridge CB2 1EW, U.K.

## Abstract

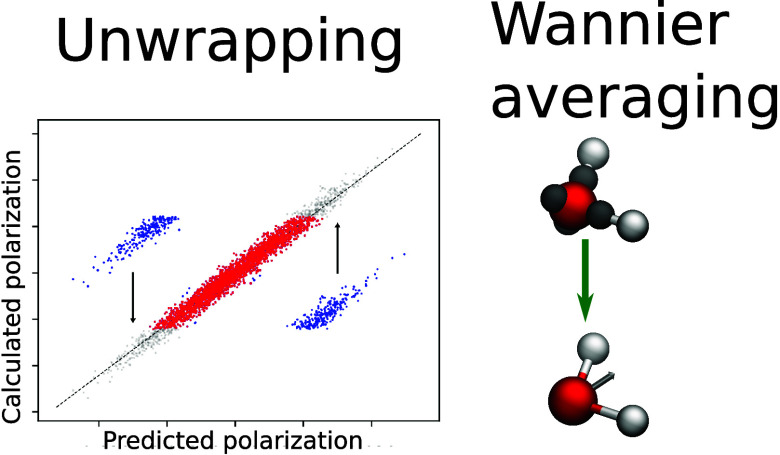

The polarization of periodically repeating systems is a discontinuous function of the atomic positions, a fact which seems at first to stymie attempts at their statistical learning. Two approaches to build models for bulk polarizations are compared: one in which a simple point charge model is used to preprocess the raw polarization to give a learning target that is a smooth function of atomic positions and the total polarization is learned as a sum of atom-centered dipoles and one in which instead the average position of Wannier centers around atoms is predicted. For a range of bulk aqueous systems, both of these methods perform perform comparatively well, with the former being slightly better but often requiring an extra effort to find a suitable point charge model. As a challenging test, we also analyze the performance of the models at the air–water interface. In this case, while the Wannier center approach delivers accurate predictions without further modifications, the preprocessing method requires augmentation with information from isolated water molecules to reach similar accuracy. Finally, we present a simple protocol to preprocess the polarizations in a data-driven way using a small number of derivatives calculated at a much lower level of theory, thus overcoming the need to find point charge models without appreciably increasing the computation cost. We believe that the training strategies presented here help the construction of accurate polarization models required for the study of the dielectric properties of realistic complex bulk systems and interfaces with ab initio accuracy.

## 1 Introduction

The polarization ***P*** of a condensed-phase system determines its coupling with an electric field, underpinning such diverse physical phenomena as linear^1^ and nonlinear spectroscopy,^2,3^ interactions between surfaces and adsorbates,^4^ and domain wall conductivity.^5,6^ To disentangle the information contained within experimental results, it is commonplace to supplement them with computer simulations.^4,7−10^ However, computing the polarization requires quantum-mechanical calculations, which can be highly sensitive to the level of theory used.^8^ Over the past few years, it has also become standard to use machine-learning (ML) methods to circumvent the need for costly quantum-mechanical calculations, with ML potentials (MLPs) for the energies and forces in a system^11−13^ and ML multipole moment surfaces (ML-MMSs) for the response to electric fields^10,14−58^ saving enormous amounts of time and effort and making it possible to tackle highly complex systems.

It is well-known that there are subtleties in the calculation of electronic polarizations in periodic systems, which due to periodic boundary conditions (PBCs) are only defined modulo a “quantum of polarization” ***Q***, proportional to the system’s unit cell.^19,20^ This means that the elements of the reduced polarization ***p*** = ***Q***^–1^***P*** are between −1/2 and +1/2. In practice, this means that ***p*** is a discontinuous function of the atomic coordinates, as a small change in positions may lead to elements of ***p*** going from ±1/2 to ∓1/2. A few methods have been given in the literature to overcome this problem and make it possible to learn the polarization: For systems containing pure water, it is possible to add integer values to ***p*** so that it agrees as well as possible with the results of a simple point charge model.^10,15,21^ However, this approach requires the availability of a reference model. Gigli et al. note that the derivatives of the polarization (i.e., the Born effective charges) can be used to render ***P*** a smooth function of atomic positions in the case of solids.^9^ Zhang et al. advocate learning the positions of Wannier centers in a system,^17^ which requires no preprocessing of data but does require that the Wannier centers are available within the data set. This approach also requires a well-defined way to assign Wannier centers to individual atoms, which may not be the case for molecules in which electrons are shared more evenly between atoms.

In this paper, we present ML models to predict the polarization of aqueous systems and compare their performance in bulk water as well as in more challenging scenarios such as the air–water interface and concentrated electrolyte solutions, in which long-ranged electrostatic effects are important. We focus on two learning strategies: In the first one, we use surrogate point charge models to render the polarization a smooth function of positions, while in the second one, we learn the positions of Wannier functions. We show that both methods lead to very accurate models for ***P***, with the former method performing slightly better for a large number of training points but requiring additional preprocessing for electrostatically complex systems. Learning the average positions of Wannier centers works better “out-of-the-box”, though only for cases where every atom of the same element is assigned the same number of Wannier centers.

We further show that even when a straightforward molecular model with point charges is not available, it is possible to generate a polarization that is a smooth function by using a data-driven approach that requires a small number of polarization derivatives with respect to atomic positions, known as Born effective charges (BEC), which are always continuous in the positions. Since the BEC can be computed at a lower level of theory than the original polarizations, even if they are not provided with the data set, their calculation requires a minor computational overhead. The remainder of this manuscript is organized as follows: In Section 2, we introduce the error function used in this work and describe the different training strategies as well as the employed data sets. In Section 3, we start by presenting the results for bulk water, followed by the study of the air–water interface, where we predict the interfacial dielectric constant and the analysis of concentrated NaCl aqueous solutions. In Section 3.4, we propose and benchmark a data-driven unwrapping procedure based on BECs, and in Section 4, we conclude the manuscript with closing remarks and a future outlook.

## 2 Computational Details

### 2.1 Error Functions for Periodic Data

When all the predicted polarizations are very similar to the target values modulo the quantum of polarization, we can use the root mean squared error (RMSE), which will allow comparison with other methods in the literature. That is,
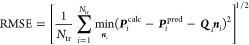
1where ***P***_*i*_^calc^ is the calculated polarization for the *i*th training structure, ***P***_*i*_^pred^ is the predicted value, ***Q***_*i*_ is the quantum of polarization for this structure, and ***n***_*i*_ is a vector containing three integers. The notation “min_***n***_*i*__” indicates that the vector ***n***_*i*_ minimizing each term should be taken.

However, in the general case, we note that a more appropriate error function for gauging predictions of circular data is the von Mises function,^22^
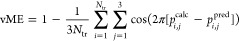
2where *N*_tr_ is the number of training points, *p*_*i*, *j*_^calc^ is the *j*th Cartesian component of the calculated ***p*** for testing structure *i*, and *p*_*i*, *j*_^pred^ is the prediction of a model. The von Mises error is unchanged when any integer *n*_*i*,*j*_ is added to *p*_*i*, *j*_^calc^, reflecting the fact that the components of ***p*** are only defined up to an additive integer.

### 2.2 Preprocessing Polarization Data

For systems that can be described well as neutral molecular units, it is possible to find a set of integers ***n***_*i*_ for each training point *i* such that a small change in the positions leads to a small change in the polarization. The polarization for these systems can be approximated straightforwardly as the sum of the molecular dipole moments ***P***^mol^ = ∑_*j*=1_**μ**_*j*_, where **μ**_*j*_ is the dipole moment of the *j*th molecule. Each individual dipole moment is a smooth function of atomic positions, meaning that ***P***^mol^ is also a smooth function. We then preprocess the data by finding the vector of integers ***n***_*i*_ for the *i*th frame such that
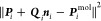
3is minimized, where ***P***_*i*_ is the calculated polarization for the *i*th frame and ***P***_*i*_^mol^ is the sum of molecular dipole moments. By making the replacement ***P***_*i*_ → ***P***_*i*_ + ***Q***_*i*_***n***_*i*_, we render the polarization a smooth function of the atomic positions while retaining the correct physics. This approach was used to build the μH_2_O model,^21^ which has been used to accurately model linear and nonlinear vibrational spectra of pure water.^10,21,23^

Since the only requirement of a reference model for the molecular dipole moments is that the prediction ***P***_*i*_^mol^ should correlate well with the unwrapped ***P***_*i*_ + ***Q***_*i*_***n***_*i*_, we are free to choose a model based on simple point charges. Since a fairly wide range of atomic charges will lead to the same set of integers ⟨***n***_*i*_⟩, we expect the models to be quite robust to the exact values used for the atomic charges; we will test this robustness along with the method. In some cases, we will also use the prediction of the reference model as a baseline to be subtracted from the training data. Once the polarizations have been processed, they can be learned with the symmetry-adapted Gaussian process regression (SA-GPR) approach,^15,24^ in which the polarization of a bulk system is predicted as a sum of atom-centered dipole contributions. In this work, we will use two different types of vector kernel as appropriate: For all systems, we will use the standard short-ranged λ-SOAP (smooth overlap of atomic positions) kernel, which is a generalization of the SOAP kernel^25^ to learn spherical tensors of order λ = 1. In cases where the polarization may be affected by long-ranged effects, we will also use the λ-LODE (long-range density equivariant) kernel,^26^ which generalizes the LODE kernel^27^ and includes nonlocal information into the learning process.

### 2.3 Learning Wannier Displacement

The polarization of a bulk system is given formally by^20^
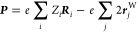
4where ***R***_*i*_ is the position of the *i*th atom, *eZ*_*i*_ is its nuclear charge, and ***r***_*j*_^W^ is the position of the *j*th Wannier center, where it is assumed that all Wannier orbitals are fully occupied. If the Wannier centers can be assigned to atoms, then
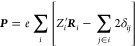
5where ∑_*j*∈ *i*_ refers to a sum over all centers assigned to atom *i*, **δ**_*ij*_ = ***r***_*j*_^W^ – ***R***_*i*_ is the displacement from the *i*th nucleus to the *j*th Wannier center, and *Z*_*i*_*′* = *Z*_*i*_ – ∑_*j*∈ *i*_ 2. An alternative model for the polarization, as suggested by Zhang et al., is to predict **Δ**_*i*_ = ∑_*j*∈ *i*_**δ**_*ij*_ for each atom to which Wannier centers are assigned.^17^ We train models for this property using standard SA-GPR, where the training set consists of local environments and their respective **Δ**_*i*_ values, and predictions are made for a new local environment using its λ = 1-SOAP kernel with the environments in the training set. ***P*** can then be predicted for a system via eq 5, using the **Δ**_*i*_ predicted for each environment and the *Z*_*i*_*′*, which can be computed once the number of Wannier centers assigned to each atom is known.

In ref (17), the **Δ**_*i*_ is predicted using neural networks; here, we predict the individual atomic **Δ**_*i*_ using SA-GPR. For the systems we will consider in this paper, the number of Wannier centers *N*_*i*_^W^ = ∑_*j*∈ *i*_ assigned to atom *i* is always either 0 (in the case of H atoms) or 4 (for heavier atoms). In future work focusing on more complex systems, such as those in which there is covalent bonding between atoms that have a more even share of electron density than does the O–H bond, the number of Wannier centers assigned to an atom may depend not only on the atom’s identity but also on its local environment.

### 2.4 Data Set Details

The models we generate in this paper are described by three strings, one describing the type of system, one the type of data that was learned, and one the descriptor type. The type of system is one of,

- bulk water (H_2_O–B),
- interfacial water (H_2_O–I), and
- concentrated sodium chloride solution (NaCl);

the type of data learned is one of,

- data preprocessed using a point charge model (PP),
- data preprocessed using a point charge model, with additional data on molecular dipole moments included during training (PP-M),
- data preprocessed using a point charge model and then baselined against the prediction of the model (PP-B), and
- average displacement of Wannier centers (W);

and the type of descriptor is one of,

- short-range λ-SOAP descriptor (SR) and
- long-range λ-LODE descriptor (LR).

Each model is then described by concatenating the three strings as SYSTEM_DATA_DESCRIPTOR; for example, H_2_O–B_PP_SR is a model trained on the preprocessed polarizations of frames of bulk water, using a short-ranged λ-SOAP descriptor. The Supporting Information gives example input files used to calculate the polarizations of these systems.

## 3 Results and Discussion

### 3.1 Bulk Water

**Figure 1:**
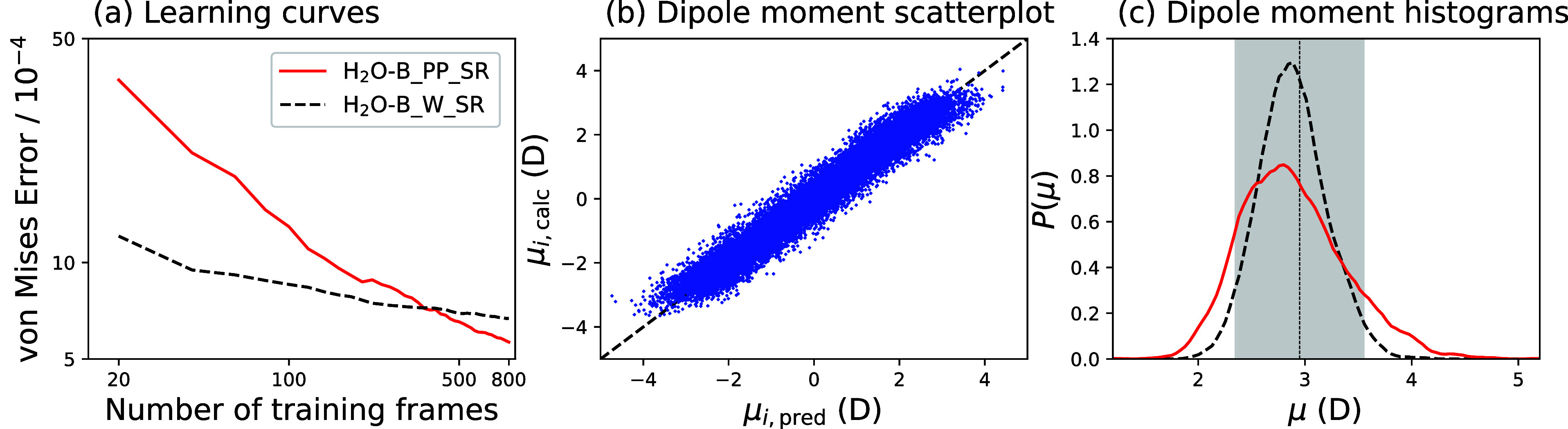
(a) Learning curves for the polarization ***P*** of bulk water systems, with the total polarization either learned directly using preprocessed data (H_2_O–B_PP_SR, solid red line) or calculated by predicting the average vector separation of Wannier centers from the oxygen atoms (H_2_O–B_W_SR, dashed black line). (b) Scatterplot of partially resummed dipole moments from the H_2_O–B_PP_SR model, comparing the prediction of the atom-centered dipole model with the *calculated* Wannier dipoles. (c) Histograms of molecular dipole moment magnitudes from calculated Wannier centers (dashed black line) and from H_2_O–B_PP_SR predictions (solid red lines). Gray shaded area shows the experimental confidence interval for the dipole moment magnitude.^33^

We began by studying pure bulk water, for which short-ranged ML models perform very well as MLPs^28−31^ and ML-MMSs.^15,17,21^ Using a set of 1000 frames each containing 32 water molecules, we tested the data preprocessing approach, for which the partial charges of the SPC/E model^32^ were used to provide molecular dipole moments. We also predicted Wannier displacements, with each Wannier center assigned to the nearest O atom; this resulted in each O atom being assigned 4 Wannier centers. Figure 1a shows learning curves of the von Mises error in predicting the polarizations of 200 bulk water frames using either the data preprocessing (H_2_O–B_PP_SR) or the Wannier displacement approach (H_2_O–B_W_SR), as a function of the number of frames used to train the model.

For a small number of training points, the H_2_O–B_W_SR model has a better performance than the H_2_O–B_PP_SR, but the error using the latter method decreases much more quickly with the number of frames, and this approach performs better for more than 500 frames. Both techniques give excellent predictions of the polarization: The corresponding RMSEs are equal to 1.3% of the intrinsic deviation in the data set for the preprocessing approach and 1.8% for the Wannier center approach. In ref (17), the average Wannier displacement assigned to the O atoms in water was learned using deep neural networks, with an RMSE of 0.032 D for the molecular dipole moments, using 95,000 training frames with 64 molecules each. Despite using ∼200 times fewer data points, our models give an RMSE of 0.053 D per molecule, indicating a very similar performance.

For the data preprocessing method, the H atoms were assigned a partial charge of *q*_H_ = +0.4238 *e*, where *e* is the electron charge, and the O atoms were assigned a partial charge of *q*_O_ = −2*q*_H_, as per the original SPC/E model.^32^ Because this processing involves rounding the elements of ***Q***_*i*_^–1^(***P***_*i*_ – ***P***_*i*_^mol^) to the nearest integer, it is possible to vary *q*_H_ between +0.3 *e* and +0.7 *e* and obtain the same training data. This suggests that the processing is extremely robust to the point charge model used and that the user is free to process their training data with whichever model is most convenient.

Given the importance of molecular dipole moments in modeling the results of experiments such as sum-frequency generation,^34−38^ it is worth investigating further the molecular dipoles that are given by the two approaches we have studied. In each case, we obtain atom-centered properties, which can be partially summed over all atoms in a molecule. Figure 1b compares the components of these molecular dipole moments from the predicted atom-centered dipole moments trained using preprocessed data with the *calculated* Wannier centers from density functional theory (DFT). Despite the fact that the model is trained on total polarizations ***P*** and the splitting into molecular dipole moments is purely data-driven, the correlation between the two is remarkably good. Histograms of the magnitudes of molecular dipole moments from the predictions and from the calculations are shown in Figure 1c: While the dipole moments predicted by SA-GPR have a broader distribution than those calculated using Wannier centers, both have their average at 2.89 D, which is within the experimental error bars.^33^ While the correlation between the dipole moments is fortunate and justifies the use of partially resummed molecular dipole predictions in calculating SFT spectra,^10^ there is no reason to believe a priori that this correlation will exist for general systems. Instead, the results of a model should be checked on a case-by-case basis before using them to make further predictions.

### 3.2 Air–Water Interface

A more challenging test of the two methods for learning polarizations is given by the air–water interface, an anisotropic slab system that contains a dielectric boundary: this means that not only are the local environments much more varied than in bulk water but also we might expect long-ranged electrostatic effects to be more important. Indeed, MLPs for aqueous interfaces that are based on short-ranged descriptors tend to give a poorer description of the spatial and orientational structure than do those in which long-ranged information is accounted for by learning the positions of partial charges.^39,40^

In Figure S1 in the Supporting Information, we show learning curves for the polarizations of water slabs, using a data set comprising 1000 frames with 128 water molecules each and with the two approaches applied to the bulk system; with the largest training set size, the von Mises error for predicting the preprocessed polarization is 7.5 × 10^–3^ (i.e., 13 times as large as for bulk water) and learning the Wannier displacement **Δ** the error is 5.4 × 10^–3^ (i.e., 8 times as large as for bulk water), albeit with an error that saturates very quickly as a function of the number of training points. In both cases, the errors are significantly larger than observed for bulk systems, likely reflecting the fact that interfacial systems are more complex, with a wider variety of local environments. In this case, the model based on learning the Wannier displacements performs slightly better, although as in the case of bulk water, the two have a very similar performance.

**Figure 2:**
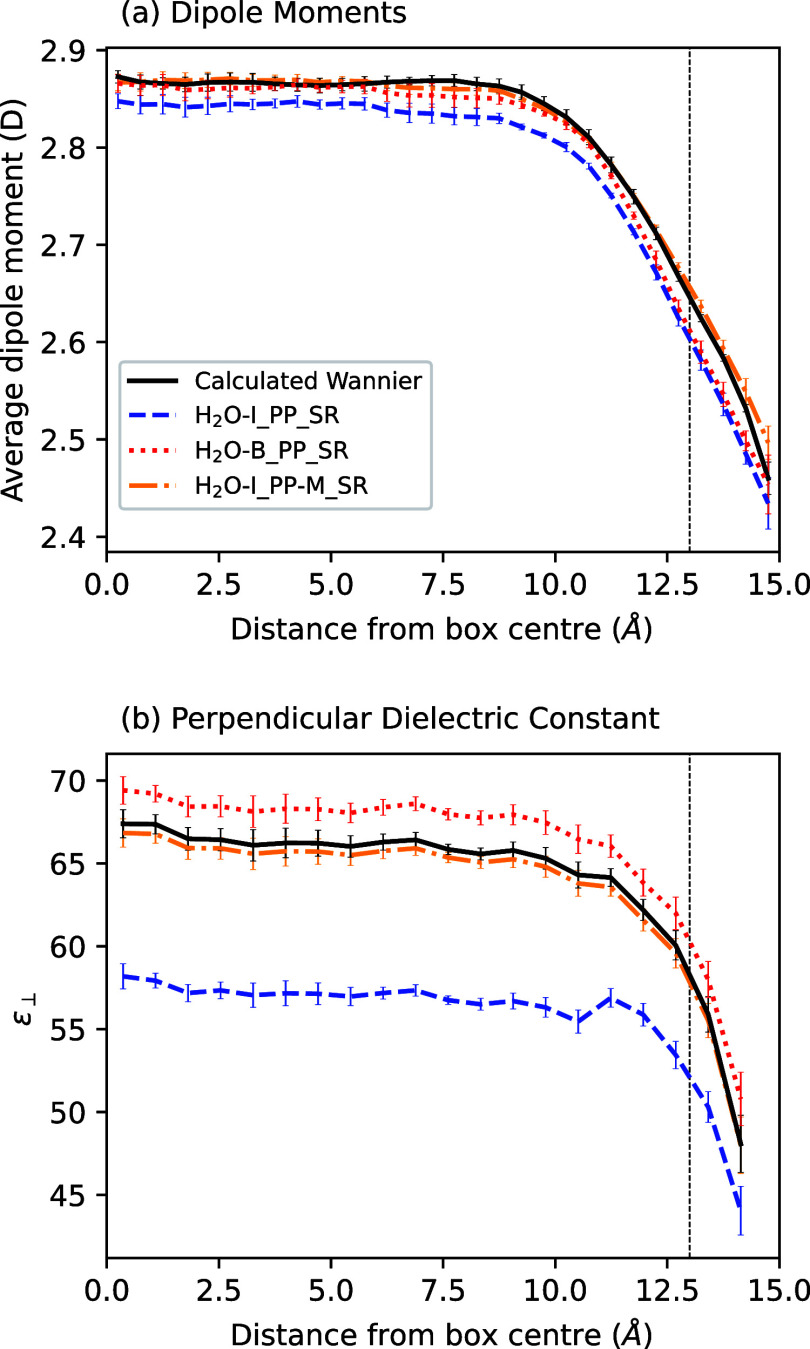
(a) Average molecular dipole moment as a function of distance from the center of a slab of water; solid black lines show the predictions from the calculated Wannier centers; dashed blue lines show the partially resummed results of SA-GPR models of total polarizations trained on water slabs (H_2_O–I_PP_SR); dotted red lines show the results of total polarization models trained on bulk water (H_2_O–B_PP_SR); and dash-dotted orange lines show the results of models trained on the total polarization of water slabs alongside molecular dipole moments calculated using point charges (H_2_O–I_PP-M_SR). Dashed vertical line shows the position of the Gibbs dividing surface. (b) Local perpendicular dielectric constant ϵ_⊥_ as a function of distance from the slab center. Here, the solid black line is computed using Wannier centers predicted from an SA-GPR model (H_2_O–I_W_SR).

Figure 2a compares the molecular dipole moments predicted by an SA-GPR model trained on the (preprocessed) total polarization of water slabs (H_2_O–I_PP_SR), with the molecular dipole moments obtained from calculating the positions of Wannier centers with DFT. In contrast to the case of bulk water, the H_2_O–B_PP_SR model gives very different molecular dipole moments than those calculated using Wannier centers. Although the dipole moment at the center of the slab is within the (large) experimental error bars,^33^ there is a statistically significant difference between the model’s predictions and the calculations, with the model trained on preprocessed data consistently giving smaller dipole moments than those predicted using Wannier centers.

In ref (10), it was shown that a model trained only on bulk water configurations gave a very good description of the SFG spectrum of the air–water interface. To gain more insight into this perhaps surprising success, we also show in Figure 2a the average molecular dipole moments for a model trained only on bulk polarizations. Indeed, the distribution of molecular dipole moments in the bulk, where the model was trained, matches very well with the Wannier center predictions; closer to the interface, this agreement is much less good. The interfacial dipole moments from the bulk polarization model are very similar to those predicted by the model trained on water slabs. In some sense, this suggests that the “best” atom-centered dipole model for the polarization of an interfacial system is one trained on the bulk phase, which performs no worse than the mode trained on water slabs at the interface, and performs better in the bulk.

To rationalize this observation, we note that the presence of dangling O–H bonds at the air–water interface means that water dipoles tend to point outward, meaning that the total polarization component normal to the interface is relatively small due to cancellation of molecular dipoles on opposite sides of the slab’s center of mass;^35,36^ this is not accounted for in our models and means that the atom-centered predictions are smaller. Figure 2a also shows the molecular dipole moment distributions from a model trained not only on the polarization of slab systems but also on the individual dipole moments of single molecules as obtained from the SPC/E model (H_2_O–I_PP-M_SR). With this simple extension, we obtain much better agreement with the molecular dipole moments from Wannier centers. In the Supporting Information, we further show that a model trained on the Wannier center displacements from bulk water (i.e., H_2_O–B_W_SR) *also* gives molecular dipole moments that do not agree with H_2_O–I_W_SR and that an accurate model for the displacements **Δ**_*i*_ of Wannier centers in slab systems must include in their training set local environments from the interface. By tuning the importance of the molecular dipole term in the fitting, we are able to build a model that predicts physically reasonable dipole moment without sacrificing the performance of the model for ***P*** on a validation set.

The performance of a model for the polarization can be further gauged by its predicted fluctuations, which are related to the dielectric constant ε_r_: In ref (18), it was shown that a model for Wannier center positions in liquid water gives an excellent description of water’s dielectric constant. We used our models to calculate the local dielectric constant ε_⊥_, resolved along the *z* direction normal to the slab,^41,42^ which further shows how well the models account for fluctuations as a function of the distance from the center of the slab. Figure 2b shows ε_⊥_ as a function of distance from the center of the box (see Supporting Information for details of the calculation), calculated along a 10 ns trajectory of SPC/E water. The calculated value of the dielectric constant depends strongly on the system size, the statistics available, and on the electrostatic constraints and boundary conditions of the simulation from which the frames were taken.^41,43^ This sensitivity, along with the fact that we have combined configurations taken from classical force field simulations with a polarization surface trained on data at the revPBE/D3 level, means that it is likely not fruitful to compare either to the experimental dielectric constant of 78 or to the value of 71 found for SPC/E water^44^ and begin simply by comparing the predictions of the four models.

As in Figure 2a, the predicted dielectric constant calculated from Wannier centers (black curve) and that from a model trained on the slab polarization along with molecular dipole moments (orange curve) is in excellent agreement. The model trained solely on slab polarizations (blue curve) predicts a much lower dielectric constant, indicating that not only are the dipole moments themselves smaller than for the other models but their fluctuations are also smaller. Finally, the predictions of the bulk model (red curve) are not in agreement with the former two in the bulk region,but are within their error bars near the interface. Overall, the consistency between H_2_O–I_W_SR and H_2_O–I_PP-M_SR together with the discrepancy from other models suggests that one of these two models should be used when modeling interfaces.

### 3.3 Concentrated Electrolyte Solutions

Electrolyte solutions contain free charges, which adds an extra layer of complexity to the problem: Opposite charges may now be separated by significant distances, and the properties of the system are determined by long-ranged electrostatics. It has been shown that accurately predicting the properties of systems containing free charges requires these long-ranged effects to be accounted for,^40,45,46^ suggesting that we may need long-ranged descriptors. We calculated the polarization ***P*** of a set of 1000 concentrated sodium chloride solution frames each containing 31 water molecules and a single NaCl pair (i.e., a concentration of ∼1.6 M). The method used to preprocess the data differs slightly from that in pure water, in which every atom could be assigned to a well-defined molecule that is expected to be almost electrically neutral.^47^ Although the NaCl solutions contain free ions, treating the Na···Cl pair as a neutral molecule allows us to calculate a target polarization ***P***^mol^ using simple point charges. For the Wannier center models, the total displacement **Δ**_*i*_ around every heavy atom (i.e., O, Na, and Cl) is found. In practice, the values of **Δ**_*i*_ for the Na and Cl centers are much smaller than for the O atoms, with the electrons much closer to the nuclei, and can be reasonably well approximated by placing the Wannier centers at the position of the nuclei without affecting the performance in predicting ***P***.

**Figure 3:**
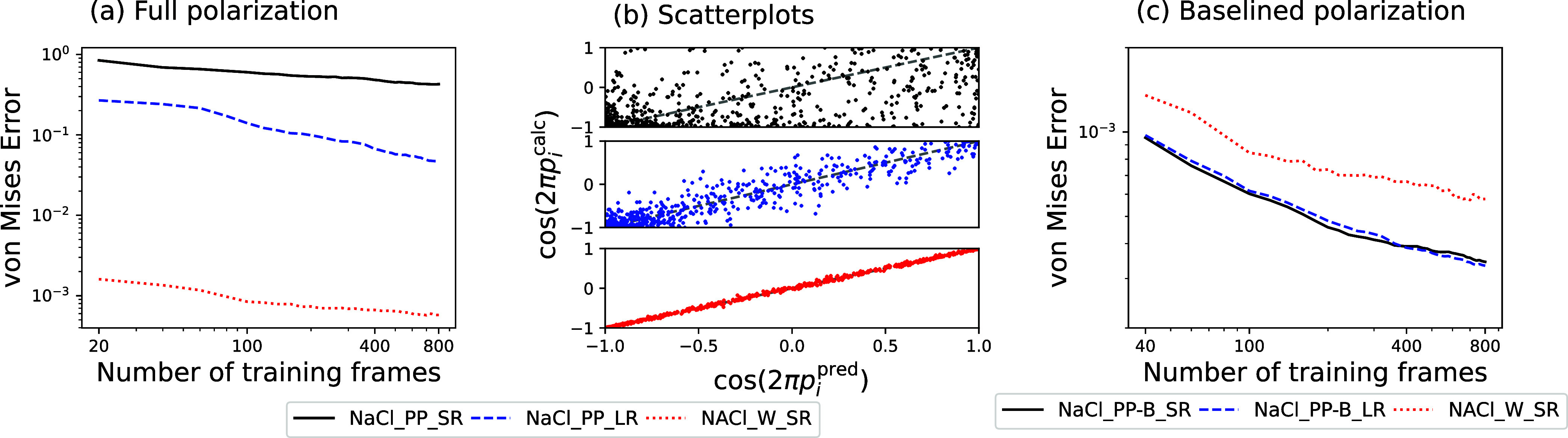
(a) Learning curves for the full polarization ***P*** of concentrated NaCl solutions, with preprocessed data learned using short-ranged λ-SOAP kernels (NaCl_PP_SR, solid black line), long-ranged λ-LODE kernels (NaCl_PP_LR, dashed blue line), or with the average displacements of Wannier centers learned (NaCl_W_SR, dotted red line). (b) Scatterplots of the predicted components of the reduced polarization ***p*** against the calculated components, with each component transformed by the cosine function to account for their circular nature. (c) As (a), but with the preprocessed data baselined against the polarization ***P***^mol^ from a point charge model (solid black line shows the results of the NaCl_PP-B_SR model and dashed blue line shows the results of the NaCl_PP-B_LR model).

Figure 3a shows learning curves for three models of the polarization of concentrated sodium chloride: two in which the polarizations are preprocessed as described above and then learned either using the short-ranged λ-SOAP kernel or a combination of the λ-SOAP and the long-ranged λ-LODE kernel and one in which the average position of Wannier centers is predicted and used to build ***P***. Scatterplots comparing the calculated reduced polarizations with the target values for the largest training set size are shown in Figure 3b. The difference between models trained using λ-SOAP and λ-LODE descriptors is significant: The short-ranged λ-SOAP model performs only a little better than drawing predictions from a uniform distribution. Incorporating long-range information with the λ-LODE models gives a much better performance, although one that is still 2 orders of magnitude worse than the performance of the models for pure water. In fact, we found that combining any amount of the λ-SOAP kernel with the λ-LODE model resulted in a worse model, with the results of Figure 3a showing the results of a pure λ-LODE model.

The difference between both of these models and the one trained on the Wannier displacements is striking: The performance of this latter model is comparable to the corresponding model for the polarization of bulk water. These results, demonstrating the importance of long-ranged information, accounted for by either allowing the partial dipole assigned to each atom to depend on the long-range structure (in the case of the preprocessed data) or explicitly accounting for charge separation via the Wannier centers, are not surprising. The improved performance for the Wannier displacement models compared to the λ-LODE model is likely due to the fact that the charge separation responsible physically for long-range electrostatic effects is built automatically into the Wannier center picture. This gives it an advantage over the λ-LODE models, which have to fit atom-centered dipoles that best model a situation where there is charge transfer between atoms.^48^

An alternative approach to account for long-ranged physics is to use baselining or delta-learning^46,49−52^ and to learn the difference between the calculated polarization and the result of another model. We thus baseline our training data against the results of the polarization model ***P***^mol^ calculated using point charges, which were readily available. This ensures that some amount of the required long-ranged physics is already present in the baseline. Figure 3c demonstrates that each of the SOAP and LODE models for the baselined polarization ***P***^Δ^ = ***P*** – ***P***^mol^ have errors 1–2 orders of magnitude lower than the models for the full polarization ***P***. The baselined SOAP model also performs better than the model trained on Wannier center **Δ**s, echoing the findings for pure water. Mixing the λ-LODE kernels with the λ-SOAP kernels led to essentially no improvement over pure λ-SOAP, indicating that the baseline point charge model already accounts for most of the long-ranged physics needed to accurately model the polarization, with short-ranged improvements made by the λ-SOAP kernel. This is underscored by the fact that the optimal weighting of the λ-LODE kernel in the combination is ∼10^–2^ times the weighting of the λ-SOAP. For concentrated solutions, we find ourselves again in the best possible situation: One in which we are free to use whichever method gives the lowest error for our data set of choice.

The simulation boxes used in this section contain a single NaCl ion pair, meaning that it is straightforward to partition the system into neutral units. A larger system containing more ions would necessitate an ion pairing scheme such as that of refs (53) and (54), which sorts oppositely charged ions into unique pairs. We are left only with the question of how to handle systems for which a simple point charge molecular model cannot be found.

### 3.4 Data-Driven Unwrapping

While the preprocessing approach we have described so far is very straightforward to implement and requires very little computational effort, it does require that a polarization model ***P***^mol^ can be found that is a smooth function of atomic positions. In turn, designing this model requires that the system is split into neutral molecular units so that ***P***^mol^ is unchanged when atoms cross periodic boundaries. For systems in which bonds can break and reform, it may not be possible to define neutral molecular units without explicitly running further calculations, which may be costly at best and highly dependent on the method used at worst.^55,56^ However, if Wannier centers are not available in a given data set or their assignment to individual atoms is ambiguous, then a way to preprocess the polarizations in a model-free manner would be required. The starting point for the approach is the fact, as pointed out by Gigli et al., that while polarizations are discontinuous, their derivatives with respect to the atomic positions, which are proportional to the BEC, *are* continuous functions of positions.^9^ By incorporating information about these derivatives into the learning process, we were able to infer the optimum preprocessing of the training data.

The method we use is as follows: we begin by training a model for polarizations, solely using the derivatives *∂ P*_*i*_/*∂ x*_*j*,*k*_ of the polarization with respect to atomic coordinates *x*_*j*,*k*_ (i.e., the *k*th Cartesian coordinate of the *j*th atom). The prediction for the *i*th component of the polarization of atomic configuration  is
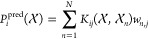
6where *N* is the number of training points (or in the case of the projected-process approach, the number of active points^57^) and  is the vector kernel between configuration  and training configuration , related to the λ = 1 kernel by a similarity transformation.^48^ The weights { ***w***_*n*_ = (*w*_*n*,1_, *w*_*n*,2_, *w*_*n*,3_, *n* = 1, ..., *N*)} are found by minimizing the loss function
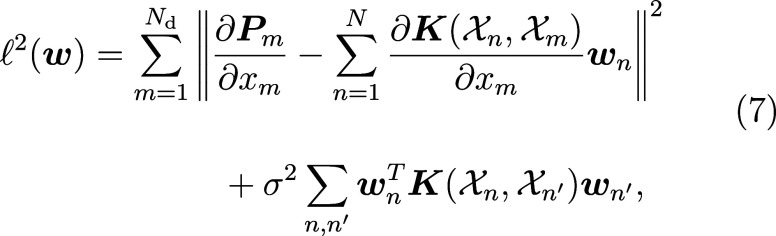
7where the index *m* refers to a training point for which we have the derivative ∂***P***_*m*_/∂*x*_*m*_ with respect to some *x*_*m*_ (a given Cartesian component of the position of a given atom in a given frame), *N*_d_ is the number of derivative training points, and  is the derivative of the kernel with respect to *x*_*m*_. σ^2^ is a regularization. By training on the derivatives of the polarization, which are a continuous function of atomic positions, the predicted polarizations are guaranteed also to be continuous functions. We have no reason to believe a priori that the predictions of this model will be accurate, and indeed, we show that they are extremely inaccurate, but there is no requirement for them to be so, as they need only to predict whether the difference in the *j*th polarization components *P*_*i*,*j*_ – *P*_*i′*,*j*_ between two frames *i* and *i′* should be positive or negative; if this prediction differs from the observed data, then we conclude that the polarization has gone through |*P*_*i*_| = *Q*_*i*_ and changed sign. By plotting the predictions of the model trained on the derivatives against the calculated values, we can identify branches in the data that allow us to add multiples of the quantum of polarization to make the data into a smooth function of position.

**Figure 4:**
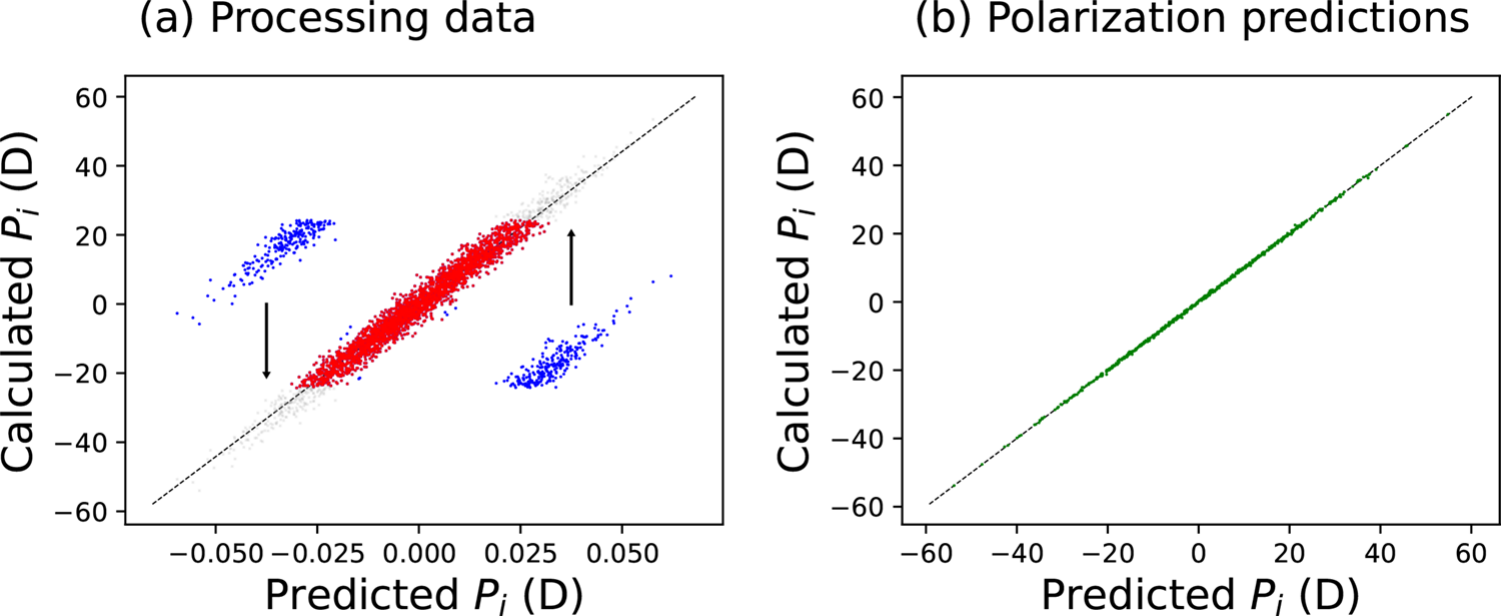
(a) Blue crosses show the total polarizations predicted using a model trained on their derivatives (eq 7). Points in red are those identified as the “main branch”, which is fit to the straight line shown. All points are then shifted by ***Qn*** to be as close as possible to the straight line, where ***Q*** is the quantum of polarization and ***n*** is a vector of integers; this is illustrated for points off of the main branch by arrows; their shifted positions are shown in gray. (b) Predictions of an SA-GPR model trained on the preprocessed data.

This is illustrated by Figure 4, which shows the results of this process as applied to pure bulk water. 800 training points were used for the derivative model, with each point the derivative of the polarization of a randomly chosen frame with respect to a randomly chosen Cartesian coordinate of a randomly chosen atom. It should be noted that this amounts to ∼0.3% of the available data. Figure 4a compares this model’s prediction of the polarization components *P*_*j*_ with the calculated values. Although they differ by several orders of magnitude, branches can clearly be identified in which there is a clear linear correlation between the predictions and the calculations. The main branch consists of data points in which the correlation can be described by a straight line through the origin, while the remaining branches are those that do not pass through the origin.

Once the points on the main branch have been identified, a straight line can be fitted through these points; each polarization is then modified as ***P***_*i*_ → ***P***_*i*_ + ***Q***_*i*_***n***_*i*_, where ***n***_*i*_ is a vector of three integers chosen to bring the result as close as possible to the straight line. In doing so, we have carried out exactly the same procedure as described in Section 2.2 and minimized the difference between our data and the results of a polarization model that is a continuous function of the atomic positions, determined in this case by a data-driven approach. The optimum values of ***n***_*i*_ for each frame are identical to those obtained using a simple point charge model, and the processed calculation data can be used to train a more accurate model as in Section 3.1, whose extremely high accuracy is illustrated in Figure 4b. Because we do not require the derivative model to give accurate results for polarizations but instead simply for the sign of polarization differences, we might expect that our results should be robust to the number of derivatives used. Using as few as 10 derivatives, we were still able to identify branches in the scatterplot between predicted and calculated polarization and thus to obtain exactly the same training data as was given by using a point charge model.

For concentrated NaCl solutions, we were unable to identify branches in the polarization data, which is unsurprising given how difficult it is to learn the pure (i.e., not baselined) polarization of this system. Rather than baselining against a point charge model for the full system, which will likely not be available if we must resort to data-driven preprocessing, we instead make the problem tractable by using a minimal amount of chemical intuition. Assigning partial charges only to the Na and Cl atoms based on their expected oxidation states in aqueous solution (+*e* and – *e,* respectively), we learn the difference between the total polarization ***P*** and the dipole moment of the Na···Cl pair. In doing so, we are able to identify distinct branches in the plot of the predictions of a derivative model against the calculated polarizations. The final model gives the same accuracy as the fully baselined results of the previous section (see Supporting Information).

Using this approach ostensibly requires that either the BEC be provided as part of the data set we would like to learn or that enough details are given on the computation of polarizations and that sufficient computer time is available to calculate their derivatives. We have, however, shown that the results are very robust to the amount of data used because it is not important for the model trained on derivatives to give an accurate polarization but merely for the predicted polarizations to be correlated with the calculated ones so that different branches can be identified. This suggests that the derivatives used in training may not need to be computed at the same level of theory as the polarizations that we would like to learn, echoing the findings of ref (9). Taking 10 frames of bulk water, choosing one Cartesian coordinate of one atom at random from each frame and calculating the derivative of the polarization with respect to this coordinate, we obtain the same preprocessed data using much looser convergence settings than the original calculation, as well as with exchange-correlation functionals that are cheaper to evaluate (see Supporting Information). Even if the BEC are not present in the data set we wish to use, it is sufficient to find a handful of polarization derivatives at whatever level of theory is most convenient and use those to infer the optimum preprocessing of the data.

### 3.5 Molecular Dynamics Trajectories

**Figure 5:**
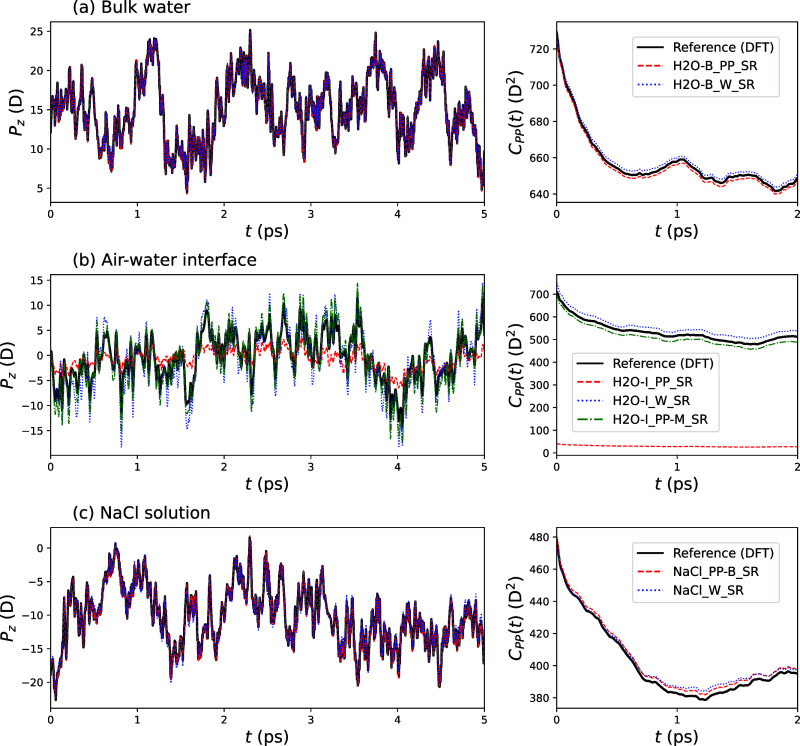
*z*-component of the system polarization along a molecular dynamics trajectory (left hand panels) and time autocorrelation function of the polarization *C*_PP_(*t*) (right hand panels) for (a) bulk water, (b) the air–water interface, and (c) concentrated NaCl solutions. In all cases, solid black lines give the results calculated using density functional theory, dashed red lines show the predictions from SA-GPR models for the postprocessed polarization (in the case of the NaCl solution, this has been baselined against the polarization from a simple point charge model), and dotted blue lines show the predictions from SA-GPR models for the Wannier displacements around each oxygen atom. Additionally, for the air–water interface, the dash-dotted green line shows the predictions of an SA-GPR model trained on the total system polarization as well as on molecular dipole moments.

To further test the performance of our models, we carried out short molecular dynamics (MD) simulations of the bulk water, the air–water interface, and concentrated NaCl solutions, using ab initio MD (AIMD) for bulk water and NaCl solutions and using the SPC/E force field for the air–water interface. For each trajectory, the polarizations were calculated using DFT calculations at the same level of theory as the training data were collected. Figure 5 compares the *z-*component of the polarization *P*_*z*_ from molecular dynamics (MD) trajectories with the results of SA-GPR models trained on preprocessed polarizations and on the Wannier center displacements. The results for the *x* and *y* components are shown in the Supporting Information. For bulk water and NaCl solutions, the postprocessed models (H_2_O-B_PP_SR and NaCl_PP-B_SR, respectively) and the models for Wannier displacements both perform excellently, giving polarizations that agree extremely well with those from DFT: Neither type of model performs noticeably better than the other. For interfacial water, while the Wannier displacement model performs extremely well, the H_2_O-I_PP_SR model underpredicts the magnitude of polarization fluctuations by a large degree. This is to be expected in light of the fact that the polarization itself was found to be smaller in this model due to symmetry considerations. The H_2_O-I_PP-M_SR model that includes information on molecular dipole moments gives agreement with the MD results that is comparable with the Wannier displacement model. The performance of all of the models is slightly worse than for the other two system types studied, reflecting the fact that the models in Section 3.2 have somewhat larger errors on their testing sets.

The polarization autocorrelation function
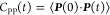
8is also shown in Figure 5, where ***P***(*t*) is the polarization vector at time *t*. *C*_PP_(0) reports on the fluctuations of the total polarization, and the *t* > 0 behavior reports on the relaxation of these fluctuations toward equilibrium. In almost every case, the agreement between the predicted and the calculated correlation function is excellent, underscoring the capability of all of these models to give very accurate results. The only exception is the autocorrelation function predicted by the H_2_O-I_PP_SR, which unsurprisingly agrees very poorly with the simulation results. However, we show in the Supporting Information that most of this discrepancy is due to the poor description of fluctuations by this model: When the correlation function is normalized by its *t* = 0 value, the time decay predicted by this model is in very good agreement with DFT results. The results in this section reinforce the point that either method of training a model for the polarizations gives results with excellent accuracy.

## 4 Conclusions

In this paper, we have presented and tested two methods for symmetry-adapted learning of the polarization of aqueous systems by either preprocessing the polarization data to obtain a suitable continuous function of atomic positions or learning the average displacement of Wannier centers from the atoms; both of these methods lead to highly accurate models. Of the two, the latter method is the most likely to work without any further modifications, requiring no specialist knowledge about the system and accounting naturally for charge separation. However, the former method has provided a slightly better accuracy for the systems studied, and crucially, this accuracy increases faster with the number of training points used. The Wannier center approach is currently applicable only to systems for which the number of centers assigned to each atom is constant; if paired with a reliable model for this number, it could be applied to more general systems.

While the excellent performance of both methods to predict total polarizations means that this added accuracy may not be needed, in situations where the Wannier center positions are difficult to learn, not provided with a data set or not straightforward to assign to atomic centers in a way that can be predicted by our models, it is practical to use a preprocessing method. Moreover, where there is no well-defined reference model that can be used to process the polarizations, this processing can instead be carried out in a data-driven way using a small number of derivatives of the polarizations with respect to atomic positions. This approach is highly robust to the level of theory used to calculate the derivatives, and therefore, a much lower level of theory can be used.

When calculating the molecular dipole moments in pure water, as required for the determination of the interfacial dielectric constant, the best results were originally obtained by predicting the positions of Wannier centers. However, by using the simple point charge model to process the raw polarizations to provide additional information to the SA-GPR model about the expected size of the molecular dipole moments, both methods were made consistent. Having demonstrated the high accuracy possible using both of these methods, future work will focus on using them to predict vibrational spectra of electrostatically complex systems such as ionic solutions and their interfaces with air.

## Data Availability

The data used to train models, as well details of quantum-mechanical calculations and the models themselves and instructions for applying them, can be found at https://github.com/dilkins/polarization-learning. The structure of this repository is described in the Supporting Information.

## Supporting Information Available

Description of online repository and further details on model building and validation (PDF)
