# Supplementary material for: Learning Electronic Polarizations in Aqueous Systems
Source: J Chem Inf Model. 2024 May 28;64(11):4426–35. doi: 10.1021/acs.jcim.4c00421 (PMC11167596; doi:10.1021/acs.jcim.4c00421)
Supplement: Supplementary file 1 — ci4c00421_si_001.pdf [file ci4c00421_si_001.pdf]

Learning Electronic Polarizations in Aqueous Systems

Arnab Jana,[†] Sam Shepherd,[†] Yair Litman,[‡] and David M. Wilkins[*,†]

†*Centre for Quantum Materials and Technologies, School of Mathematics and Physics, Queen's University Belfast, Belfast BT7 1NN, Northern Ireland, United Kingdom*

‡*Yusuf Hamied Department of Chemistry, University of Cambridge, Lensfield Road, Cambridge, CB2 1EW, UK*

E-mail: d.wilkins@qub.ac.uk

1 Calculation Details

A github repository is provided, at,

`https://github.com/dilkins/polarization-learning`,

containing files for calculations and applying the models.

All calculations were carried out using density functional theory (DFT) with the CP2K code.[1] The revPBE-D3 functional was used,[2–5] with the TZV2P-GTH basis set and Goedecker-Teter-Hutter pseudopotentials.[6] All input frames and sample input files are included in the subfolders of `calculations/` in the repository.

Symmetry-adapted Gaussian process regression (SA-GPR)[7] models were trained using the same hyperparameters used in Ref. 8 to train the μ-H_2O model. The subfolders of `models/`, in the repository, contain files to implement all of the models trained.

Further information is given in the `README.txt` files of these folders.

2 Water Slab Learning Curves

Fig. S1 shows learning curves for the polarization of the air-water interface systems using either the data pre-processing approach or by learning the positions of Wannier centres. There are several differences between these learning curves and those shown in Fig. 1(a) in the mean text for pure bulk water: most notably, the Wannier centre model performs much better compared to the pre-processed data model; as discussed in the main text, the symmetry of the water slabs biases the predicted molecular dipole moments, meaning that they are systematically smaller. This biasing is likely the reason for the relatively poorer performance.

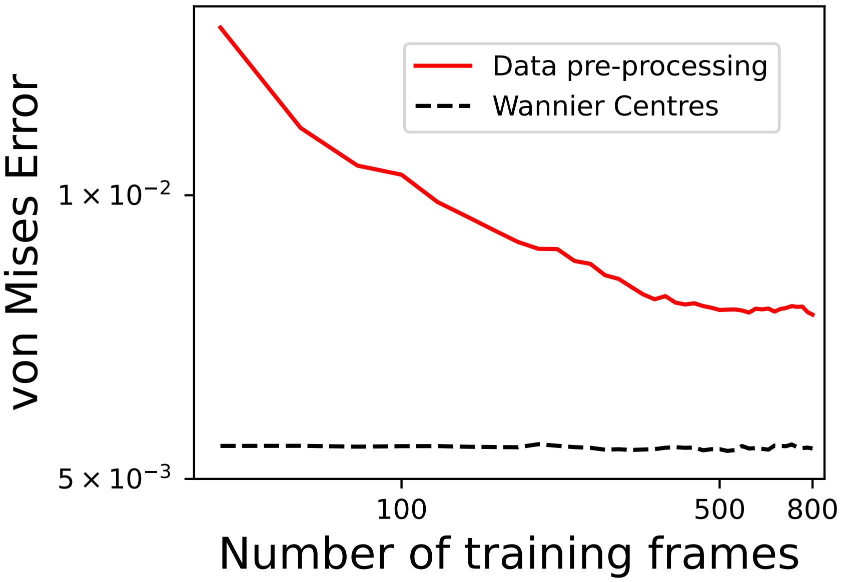

Figure S1: Learning curves for the von Mises error in predicting the polarization of water slabs as a function of the number of frames used to train the model, either by pre-processing the data using a point charge model (solid red line) or by learning the average displacement of Wannier centres from the oxygen atoms (dashed black line).

In addition, the model for average Wannier centre displacements saturates very quickly: the slab systems contain 128 water molecules, as opposed to the 32 molecules used for the

bulk model, and so the effective number of training points is proportionally larger. Given that this model is already excellent, there is little need, for our current purposes, to improve it further.

3 Models Trained on Slab Polarizations and Molecular Dipoles

The predictions of SA-GPR models trained on the polarization of a system are given by,

$$P_\alpha^{\text{pred}}(\mathcal{X}_i) = \sum_j \sum_\beta K_{\alpha\beta}(\mathcal{X}_i, \mathcal{X}_j) w_{j,\beta}, \tag{S1}$$

where $P^{\text{pred}}(\mathcal{X}_i)$ is the polarization for the system represented by $\mathcal{X}_i$, $K_{\alpha\beta}(\mathcal{X}_i, \mathcal{X}_j)$ the (α, β)-component of the vector SA-GPR kernel between systems $\mathcal{X}_i$ and $\mathcal{X}_j$, and $w_{j,\beta}$ a weight that is computed, using the projected-processes approach[9] as,

$$\boldsymbol{w} = \left(\boldsymbol{K}_{NM}^\top \boldsymbol{K}_{NM} + \sigma^2 \boldsymbol{K}_{MM}\right)^{-1} \boldsymbol{K}_{NM}^\top \boldsymbol{P}^{\text{calc}}. \tag{S2}$$

Here, $\boldsymbol{K}_{NM}$ is the kernel matrix between members of the (N-dimensional) training set and those of the (M-dimensional) active set; $\boldsymbol{K}_{MM}$ is between pairs of members of the active set. σ^2 is a regularization parameter and $\boldsymbol{P}^{\text{calc}}$ is a vector containing the polarizations of members of the training set. It should be noted that, e.g., the vectors $\boldsymbol{w}$ and $\boldsymbol{P}^{\text{calc}}$ are "vectors of vectors" (i.e., matrices), but that we use this notation as it is familiar from standard Gaussian process regression (GPR).

Eq. (S2) can be modified to include molecular dipole moments, in this case calculated using the SPC/E model,

$$\boldsymbol{w} = \left(\boldsymbol{K}_{NM}^\top \boldsymbol{K}_{NM} + \gamma \boldsymbol{K}_{PM}^\top \boldsymbol{K}_{PM} + \sigma^2 \boldsymbol{K}_{MM}\right)^{-1} \left(\boldsymbol{K}_{NM}^\top \boldsymbol{P}^{\text{calc}} + \gamma \boldsymbol{K}_{PM}^\top \boldsymbol{\mu}^{\text{calc}}\right), \tag{S3}$$

with $\boldsymbol{K}_{PM}$ the kernel between individual water molecules (of which there are P in the training set) and members of the active set, $\boldsymbol{\mu}^{\text{calc}}$ a vector containing calculated molecular dipole moments, and γ a hyperparameter that determines the relative importance of fitting to molecular dipoles compared to total polarizations. This was tuned to a value of 0.05, which gave good agreement with the molecular dipoles of a validation set, while not losing accuracy on their polarizations.

4 Wannier Centre Models on Slab Systems

Fig. S2 compares the average magnitudes of local molecular dipole moments as a function of their distance from the centre of a water slab, for the calculated Wannier centres, and `H2O-B_W_SR`, trained on Wannier displacements from bulk water systems. The discrepancy between these two results leads us to conclude that without having been trained on local environments that contain interfaces, the Wannier centre models from pure bulk simulations give results that are quantitatively incorrect.

Fig. S3 shows the average magnitudes of local molecular dipole moments as a function of their distance from the slab centre for the calculated Wannier displacements and from the Wannier displacements predicted from an SA-GPR model. The two results are in excellent agreement.

5 Local Perpendicular Dielectric Constants

As described in Olivieri *et al.*, the local dielectric constant $\varepsilon_\perp(z)$ as a function of the z position relative to the centre of the slab was given by,[10]

$$\frac{(\varepsilon_\perp(z)-1)(2\varepsilon_\perp(z)+1)}{\varepsilon_\perp(z)} = \frac{3}{\varepsilon_0 k_{\text{B}} T}\rho_N(z)\left\langle \mu_\perp^2(z)\right\rangle G_K(z, r > r_{\text{K}}), \tag{S4}$$

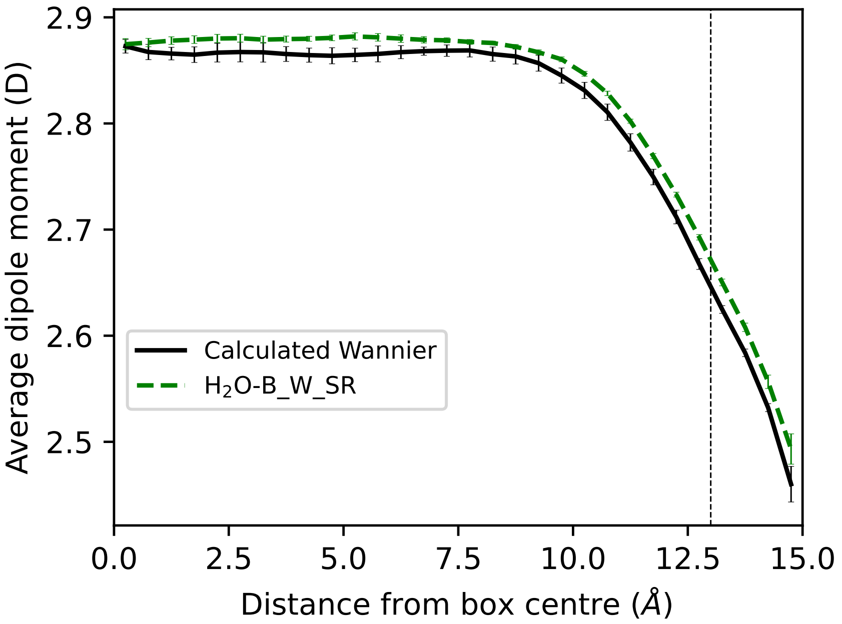

Figure S2: Average molecular dipole moment as a function of distance from the centre of a slab of water; solid black lines show the predictions using the calculated Wannier centre displacements, and dashed green lines the predictions of a model trained on bulk data (`H2O-B_W_SR`). The dashed vertical line shows the position of the Gibbs dividing surface.

with ε_0 the free space permittivity, k_B Boltzmann's constant and T the temperature, $\rho_N(z)$ the local density as a function of distance from the slab centre, $\langle \mu_\perp(z)^2 \rangle$ the mean square projection of the molecular dipole moment on the z axis (i.e., normal to the slab) and,

$$G_K(z,r) = \left(\langle \mu_{\perp,1} M_{\perp,1}(r) \rangle_{z_1=z} - \langle \mu_{\perp,1} \rangle_{z_1=z} \langle M_{\perp,1}(r) \rangle_{z_1=z} \right) / \left\langle \mu_\perp(z)^2 \right\rangle, \tag{S5}$$

with $\mu_{\perp,1}$ the perpendicular component of the dipole moment of molecule 1 and $M_{\perp,1}(r)$ the total dipole moment of a sphere of molecules of radius r surrounding molecule 1 (including the central dipole moment). The average $\langle \cdots \rangle_{z_1=z}$ indicates that the z_1 coordinate of molecule 1 is constrained at z. r_K, the Kirkwood length, is a distance after which $G_K(z,r)$ reaches a plateau.

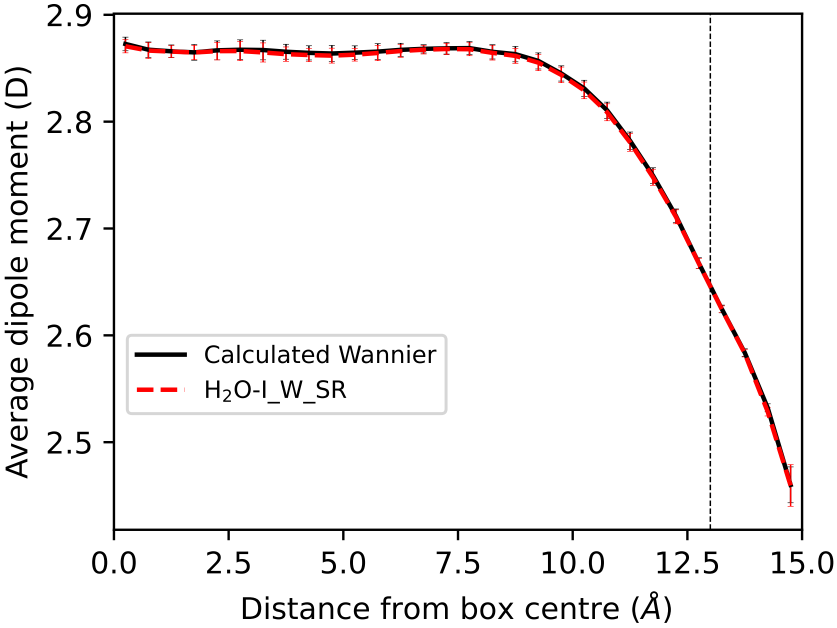

Figure S3: Average molecular dipole moment as a function of distance from the centre of a slab of water; solid black lines show the predictions using the calculated Wannier centre displacements, while dashed green lines give the predictions of a model trained on the Wannier centre displacements ($\texttt{H}_2\texttt{O-I\_W\_SR}$). The dashed vertical line shows the position of the Gibbs dividing surface.

6 Electrolyte Solution Molecular Dipole Moments

Fig. S4(a) shows the scatterplot of molecular dipole moment components calculated using Wannier centres against those predicted using models trained on pre-processed data, baselined against the point charge polarization. As for the molecular dipole moments of bulk water, the correlation between the two predictions is excellent. This is further underscored by Fig. S4(b), which gives the histograms of molecular dipole moment magnitudes: the distribution of Wannier centre dipole moments is broader, as before, but the positions of the maxima are in very agreement. These results show that it is possible to use SA-GPR to build polarization models that give an excellent reproduction of the local dipole moments, even in a system where the electrostatics are more complex than in pure water.

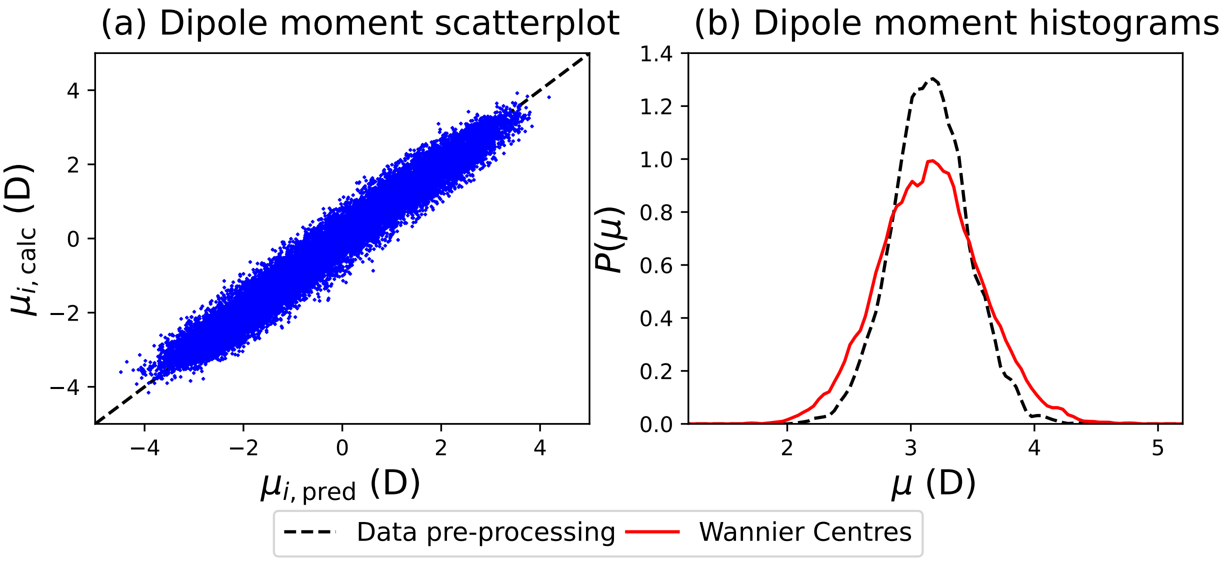

Figure S4: (a) Scatterplot of partially resummed dipole moments from the NaCl_PP-B_SR model, comparing the prediction of the atom-centred dipole model with the *calculated* Wannier dipoles. (b) Histograms of molecular dipole moment magnitudes from calculated Wannier centres (solid black lines) and from NaCl_PP-B_SR predictions (dashed red lines).

7 Data-Driven Unwrapping for Electrolyte Solutions

Fig. S5 illustrates the data-driven pre-processing approach for concentrated NaCl solutions. This can be compared directly to Fig. 4 in the main text, for bulk water: the data-driven method works for both of these systems.

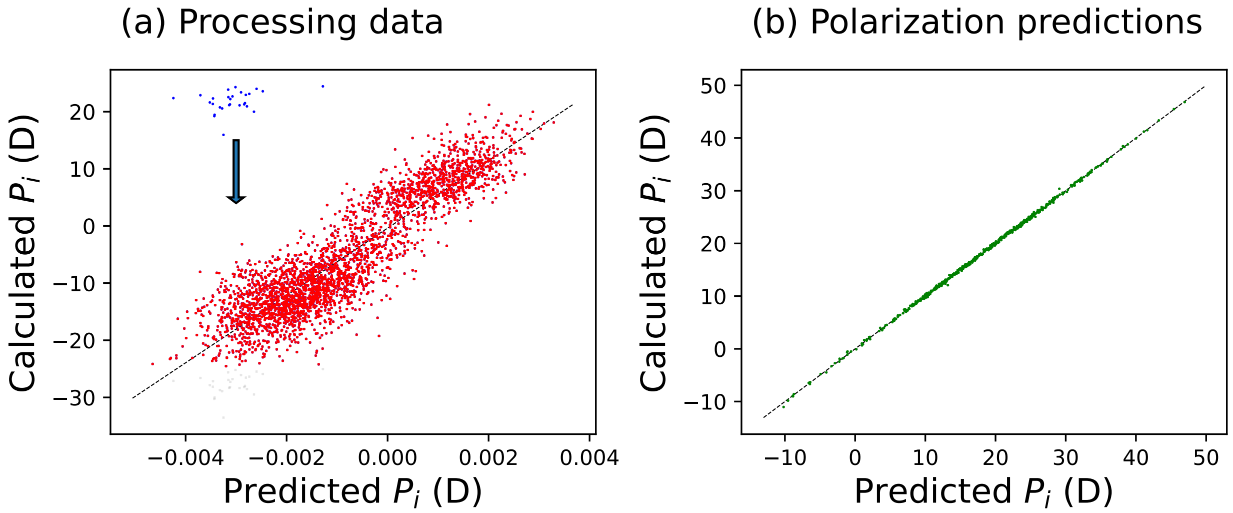

Figure S5: (a) Blue crosses show the total polarizations predicted using a model trained on their derivatives. The points in red are identified as the "main branch", which is fit to a straight line. All points are then shifted by $\boldsymbol{Qn}$ to be as close as possible to the straight line, with $\boldsymbol{Q}$ the quantum of polarization and $\boldsymbol{n}$ a vector of integers. (b) Predictions of an SA-GPR model trained on the pre-processed data.

8 Robustness of Data-Driven Unwrapping to Level of Theory

For the data-driven pre-processing of bulk water polarizations, the data shown in Fig. 4 of the main text come from a model trained on polarization derivatives calculated at the same level of theory as the polarizations themselves. We made the following changes sequentially to the input files, and at all steps the pre-processed data remained identical to that obtained from comparison to a point charge model as in section IIIA of the main text:

1. Increasing the `EPS_SCF` parameter for self-consistent field convergence from 10^{-7} to 10^{-4} and the finest cutoff from 300 Ry to 100 Ry.

2. Not using D3 dispersion corrections.

3. Replacing the revPBE functional with the LDA functional.

4. Further increasing the `EPS_SCF` parameter to 10^{-2}.

5. Replacing the TZV2P-GTH basis function with the SZV-GTH.

The data-driven unwrapping is thus extremely insensitive to the level of theory used to calculate the polarization $\boldsymbol{P}$.

9 Further Details on MD Comparisons

Fig. S6 shows the comparison of the x and y components of the polarization along a molecular dynamics trajectory with the predictions of the same models used in the main text, for bulk water, the air-water interface and concentrated NaCl solutions. The results are very similar to those in Figure 5 of the main text, indicating that the conclusions drawn when focussing on the P_z component are universally applicable. In Fig. S7 we also show the polarization autocorrelation function $C_{PP}(t)$ (Eq. 8 in the main text), divided by its value at $t = 0$:

this normalizes out the ability of the models to correctly describe the fluctuations of the polarization, and shows instead only their ability to describe how these fluctuations relax towards equilibrium. Although here the $\mathtt{H}_s\mathtt{O\text{-}I\_PP\_SR}$ still performs worst for the air-water interface, the discrepancy is much less pronounced than in the main text, suggesting that the main problem with this model is its description of the magnitude of the fluctuations, rather than the dynamics, of the polarization.

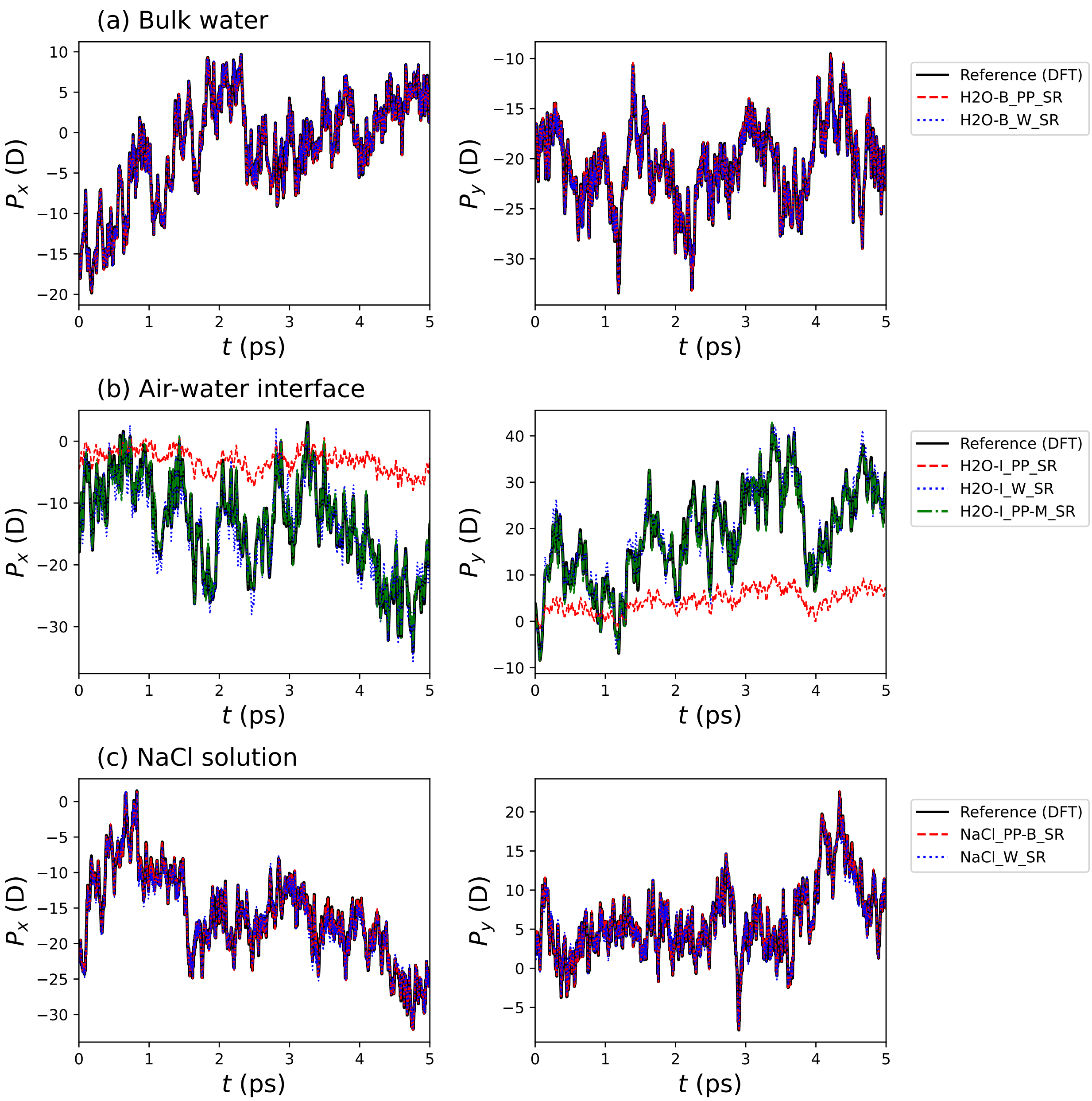

Figure S6: x-component (left-hand panels) and y-component (right-hand panels) of the system polarization along a molecular dynamics trajectory for (a) bulk water, (b) the air-water interface and (c) concentrated NaCl solutions. In all cases, solid black lines give the results calculated using density functional theory (DFT), dashed red lines the predictions from SA-GPR models for the post-processed polarization (in the case of the NaCl solution, this has been baselined against the polarization from a simple point charge model), and dotted blue lines the predictions from SA-GPR models for the Wannier displacements around each oxygen atom. Additionally, for the air-water interface the dash-dotted green line shows the predictions of an SA-GPR model trained on the total system polarization as well as on molecular dipole moments.

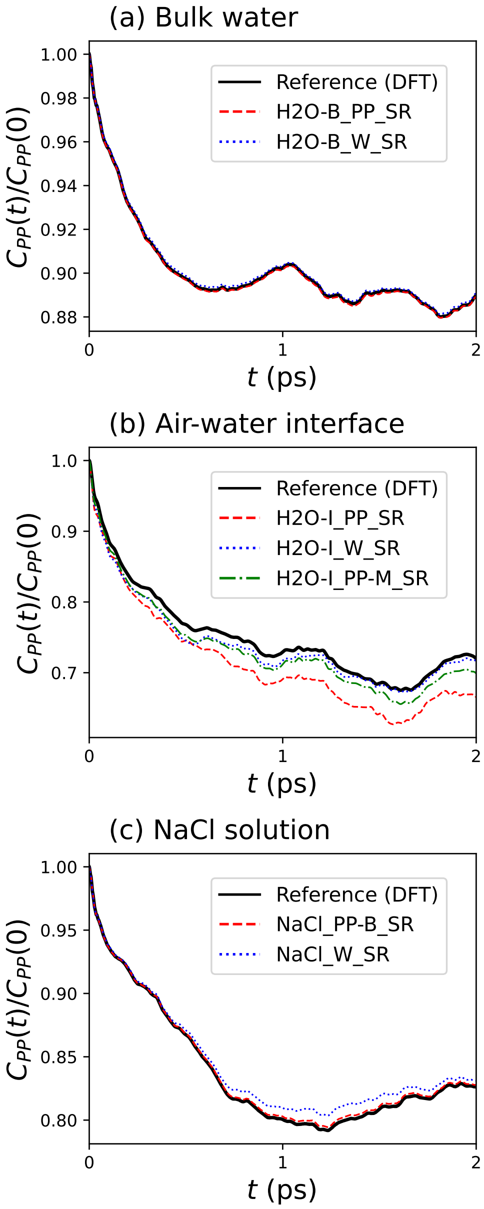

Figure S7: Polarization autocorrelation function $C_{PP}(t)$, normalized by its value at $t = 0$ for (a) bulk water, (b) the air-water interface and (c) concentrated NaCl solutions. In all cases, solid black lines give the results calculated using density functional theory (DFT), dashed red lines the predictions from SA-GPR models for the post-processed polarization (in the case of the NaCl solution, this has been baselined against the polarization from a simple point charge model), and dotted blue lines the predictions from SA-GPR models for the Wannier displacements around each oxygen atom. Additionally, for the air-water interface the dash-dotted green line shows the predictions of an SA-GPR model trained on the total system polarization as well as on molecular dipole moments.

References

(1) Kühne, T. D.; Iannuzzi, M.; Del Ben, M.; Rybkin, V. V.; Seewald, P.; Stein, F.; Laino, T.; Khaliullin, R. Z.; Schütt, O.; Schiffmann, F. *et al.* CP2K: An electronic structure and molecular dynamics software package - Quickstep: Efficient and accurate electronic structure calculations. *J. Chem. Phys.* **2020**, *152*, 194103.

(2) Perdew, J. P.; Burke, K.; Ernzerhof, M. Generalized Gradient Approximation Made Simple. *Phys. Rev. Lett.* **1996**, *77*, 3865.

(3) Zhang, Y.; Yang, W. Comment on "Generalized Gradient Approximation Made Simple". *Physical Review Letters* **1998**, *80*, 890–890.

(4) Grimme, S.; Antony, J.; Ehrlich, S.; Krieg, H. A consistent and accurate ab initio parametrization of density functional dispersion correction (DFT-D) for the 94 elements H-Pu. *J. Chem. Phys.* **2010**, *132*, 154104.

(5) Grimme, S.; Ehrlich, S.; Goerigk, L. Effect of the damping function in dispersion corrected density functional theory. *J. Comp. Chem.* **2011**, *32*, 1456.

(6) Goedecker, S.; Teter, M.; Hutter, J. Separable dual-space Gaussian pseudopotentials. *Phys. Rev. B* **1996**, *54*, 1703.

(7) Grisafi, A.; Wilkins, D. M.; Csányi, G.; Ceriotti, M. Symmetry-Adapted Machine Learning for Tensorial Properties of Atomistic Systems. *Physical Review Letters* **2018**, *120*, 036002.

(8) Kapil, V.; Wilkins, D. M.; Lan, J.; Ceriotti, M. Inexpensive Modelling of Quantum Dynamics using Path Integral Generalized Langevin Equation Thermostats. *J. Chem. Phys.* **2020**, *152*, 124194.

(9) Rasmussen, C. E.; Williams, C. K. I. *Gaussian Processes for Machine Learning*; MIT Press: London, 2005.

(10) Olivieri, J.-F.; Hynes, J. T.; Laage, D. Confined Water's Dielectric Constant Reduction Is Due to the Surrounding Low Dielectric Media and Not to Interfacial Molecular Ordering. *J. Phys. Chem. Lett.* **2021**, *12*, 4319.
